# Case Report: Localized neutrophilic dermatosis at a split-skin donor site during PD-1 blockade: a unique immune-related adverse event

**DOI:** 10.3389/fimmu.2026.1730549

**Published:** 2026-07-01

**Authors:** Veselina Moravenova, Raphael Wilhelm, Jakob Veeser, Klajdi Begaj, Beate Weidenthaler-Barth, Stephan Grabbe, Henner Stege, Miriam Wittmann

**Affiliations:** 1 Department of Dermatology, University Medical Center of the Johannes Gutenberg University, Mainz, Germany; 2 Research Center for Immunotherapy (FZI), Medical Center of the Johannes Gutenberg University Mainz, Mainz, Germany

**Keywords:** immune checkpoint inhibitors, immune-related adverse events, malignant melanoma, neutrophilic dermatoses, pyoderma gangrenosum, wound healing disorder

## Abstract

The prognosis of melanoma has significantly improved since the introduction of immune checkpoint inhibitor (ICI) therapies. While ICIs are associated with a range of immune-related adverse events (irAEs), these reactions may occur early or late during treatment. Neutrophilic dermatoses, including pyoderma gangrenosum, are commonly observed in patients with systemic inflammatory disease, but have also been reported as rare cutaneous irAEs. To further characterize this rare phenomenon, we present the case of a 45-year-old patient with stage IIB cutaneous melanoma who underwent surgical resection of the primary tumor with split-thickness skin graft coverage, followed by adjuvant therapy with the PD-1 inhibitor nivolumab. During the one-year course of immunotherapy, the donor site of the graft exhibited persistent non-healing and progressive ulceration. However, it did not extend beyond the boundaries of the initial harvest site. Of note, the recipient site at the left heel healed without complications. The lesion was biopsied after completion of immunotherapy. Histopathological analysis revealed a neutrophilic dermatosis with PG-like features. The lesion showed rapid improvement upon initiation of dapsone therapy, which is in line with its action on neutrophils. This case underscores the diagnostic challenge posed by atypical cutaneous irAEs, particularly when localized and lacking systemic features. Awareness of such rare presentations is crucial to enable timely recognition and appropriate treatment.

## 1 Introduction

Immune checkpoint inhibitors (ICIs) have transformed the field of modern oncology, offering improved survival rates, particularly in melanoma (1). Since their introduction, numerous clinical trials have demonstrated favorable outcomes, leading to their widespread use across multiple cancer types (2). However, ICIs are also associated with a broad spectrum of immune-related adverse events (irAEs), which can affect any organ system (3).

Common irAEs include skin reactions (e.g. rash, pruritus, vitiligo), gastrointestinal side effects (e.g. colitis), endocrinopathies (e.g. thyroiditis, hypophysitis) and hepatotoxicity (4). By contrast, rare but clinically significant manifestations, such as neutrophilic dermatoses, are less well-characterized and can present diagnostic and therapeutic challenges if not promptly recognized (5).

Neutrophilic dermatoses are a heterogeneous group of autoinflammatory skin disorders characterized histologically by dense infiltration of mature neutrophils in the absence of infection (6). Among them, pyoderma gangrenosum (PG) has been reported in association with ICI therapy in isolated cases (7, 8). Importantly, immune checkpoint blockade not only enhances T-cell activity but also contributes to neutrophil-driven inflammation through indirect mechanisms, including altered cytokine and chemokine signaling (9). PG may also occur spontaneously or be triggered by cutaneous trauma as a pathergy phenomenon. It typically responds well to systemic corticosteroids and immunosuppressive therapy but often worsens with surgical intervention due to pathergy reaction (10, 11).

Here, we describe a unique presentation of an ICI-associated neutrophilic dermatosis with features resembling PG, but with atypical clinical presentation and evolution, in a patient undergoing immunotherapy with nivolumab for melanoma. Nivolumab, a programmed death-1 (PD-1) inhibitor, approved for use in melanoma as adjuvant immunotherapy, has been associated with various cutaneous immune-related adverse events, including rare cases of neutrophilic dermatoses like PG (8, 12). However, these can also occur independently of an ICI-therapy, making it essential to differentiate between paraneoplastic, idiopathic, and PD-1/CTLA-4 inhibitor–associated etiologies (13). In ICI-associated cases, the clinical course is often more resistant to corticosteroid therapy alone and may require additional immunosuppressive and immunomodulating agents (10).

## 2 Case presentation

A 45-year-old male presented at our skin cancer clinic in February 2024 for excision of an acral lentiginous melanoma of the left heel. The tumor had a Breslow thickness of 4.2 mm without ulceration, corresponding to a TNM stage of pT4a and stage IIB based on AJCC criteria. Surgical excision was performed with 2 cm margins.

**Figure 1:**
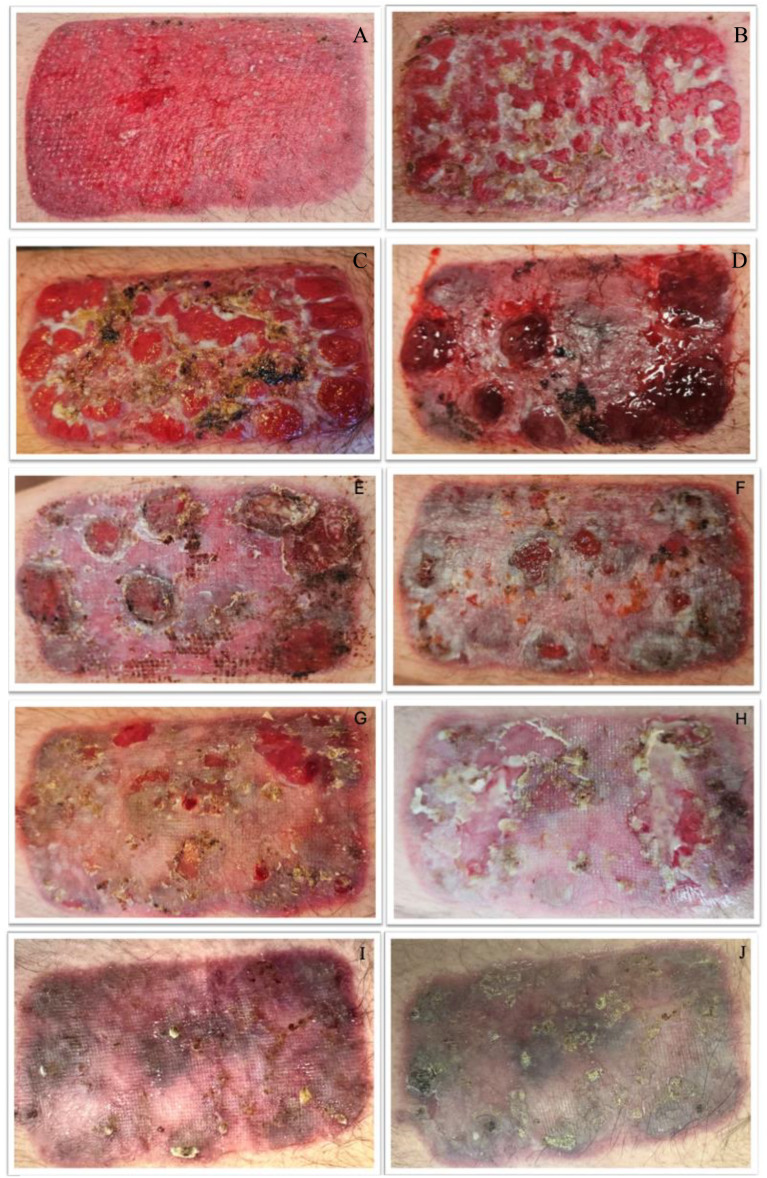
Clinical course of the wound. **(A)** 03/2024 - Immediately after skin grafting, initial tendency toward healing. **(B)** 05/2024 - Onset of exudation following the first cycle of ICI. **(C)** 05/2024 - Worsening of local findings. **(D)** 07/2024 - Temporary discontinuation of ICI due to further deterioration. **(E)** 09/2024 - Clinical improvement after an 11-week treatment pause. **(F)** 09/2024 - Re-flare with deterioration of local findings after re-initiation of ICI. **(G)** 11/2024 - Stabilization of clinical condition. **(H)** 05/2025 - Recurrence of exudation and local bleeding. **(I)** 06/2025 - Marked improvement of the lesion with Dapsone treatment. **(J)** 07/2025 - Sustained clinical improvement.

The resulting defect was reconstructed with a split-thickness skin graft (STSG) harvested from the patient’s right thigh. In the immediate postoperative period, both the graft and donor sites initially demonstrated satisfactory healing (**Figure 1A**). Additionally, a sentinel lymph node was removed during surgery and tested negative for metastatic involvement.

**Figure 2:**
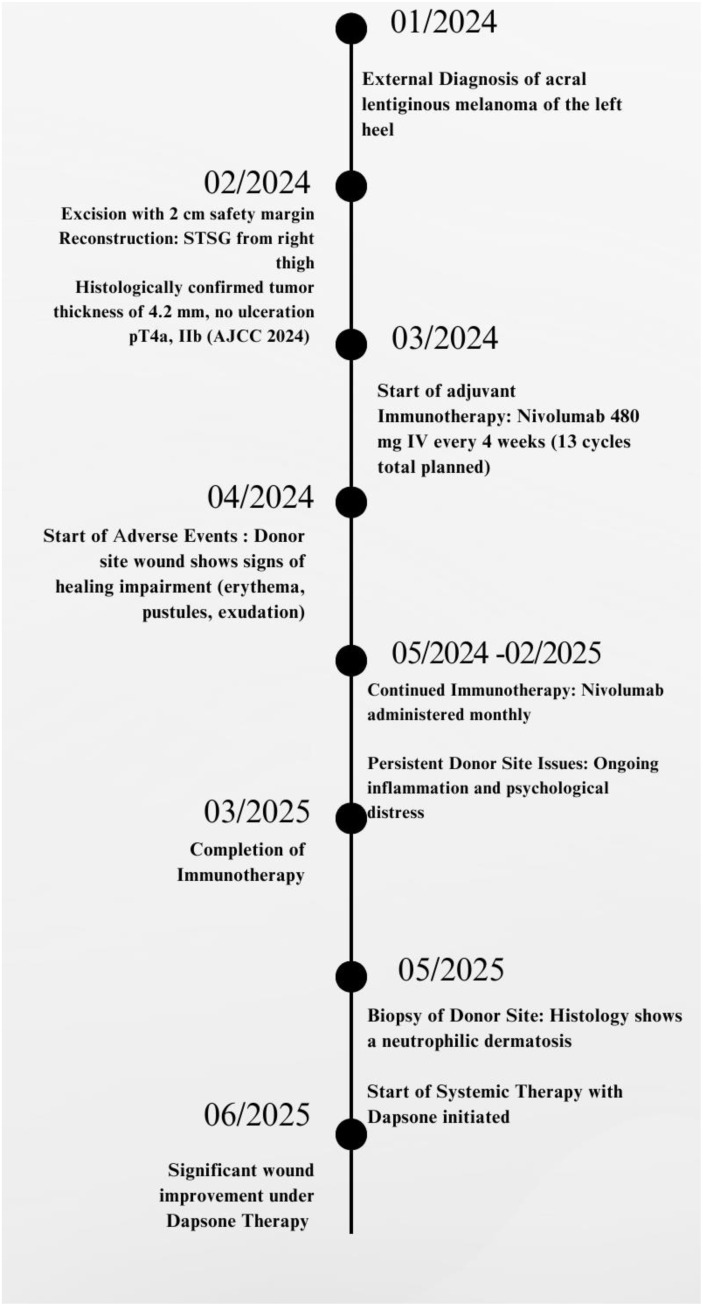
Timeline illustrating the clinical course of the patient, including key diagnostic steps, therapeutic interventions, and follow-up milestones. STSG, Split-thickness skin graft.

Based on the patient’s age and final stage T4a pN0(sn) cM0, an adjuvant immunotherapy with the anti-PD-1 agent Nivolumab (480 mg, every 4 weeks for 1 year) was initiated according to decision in our multidisciplinary tumor board (**Figure 2**).

While the skin graft at the heel healed uneventfully, the donor site developed progressive wound healing impairment (**Figure 1B**). Clinical findings included a sharply demarcated erythematous area with pustules, erosions, and persistent exudation (**Figures 1C, D**). The patient reported consistent worsening of the wound one day after each Nivolumab infusion, with no other triggers identified. This pattern persisted throughout the treatment course, contributing to significant psychological distress and quality of life impairment. The immunotherapy regimen was completed in March 2025 after a total of 13 cycles. Topical wound care as well as local corticosteroid therapy were attempted but only resulted in temporary and incomplete improvement (**Figures 1E–H**). There were no clinical signs of infection, and swab cultures remained unremarkable. Additional histochemical workup included Alcian-PAS staining, which showed no evidence of fungal elements, and direct immunofluorescence, which was unremarkable, effectively excluding immune-complex-mediated and infectious etiologies. Laboratory investigations, including inflammatory markers and infectious serologies, were non-specific. Complete blood counts and renal and hepatic function tests remained within normal limits.

**Figure 3:**
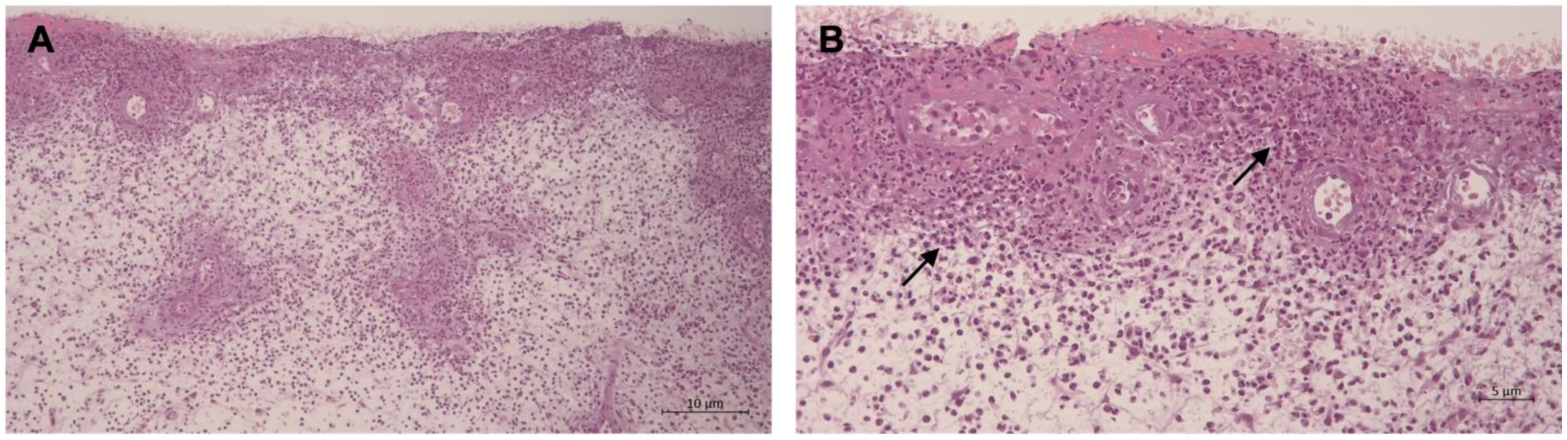
Histological findings. **(A)** 100x magnification: Neutrophilic infiltrate diffusely present throughout the dermis, without vessel wall damage. **(B)** 200x magnification: Compact collections of neutrophils (arrows indicating representative foci of dense neutrophilic infiltrate), no evidence of vasculitis or atypia.

Given the chronicity and atypical nature of the lesion, a diagnostic biopsy of the donor site was performed after completion of the immunotherapy course, as the clinical diagnosis remained uncertain throughout the treatment period. Histopathologic examination revealed a dense neutrophilic infiltrate without evidence of vasculitis or malignancy, consistent with a localized neutrophilic dermatosis with PG-like features. Classic pyoderma gangrenosum and erosive pustular dermatosis (EPD) were considered in the differential diagnosis but could not be fully confirmed given the atypical clinical morphology (**Figure 3**).

The differential diagnosis was considered systematically. Delayed wound healing was unlikely given the sharply demarcated inflammatory morphology and absence of healing trajectory despite optimal wound care. Bacterial superinfection was excluded by consistently negative swab cultures. Contact dermatitis was not supported by the clinical history or distribution. Classic PG was considered but the absence of characteristic undermined ulceration and violaceous borders made a definitive diagnosis difficult. EPD is predominantly a disease of the scalp in elderly patients following trauma (14). Drug-related eruption attributable to agents other than Nivolumab was not evident from the clinical history. Vascular compromise was excluded by the absence of peripheral pulse abnormalities and the clinical context. Melanoma recurrence or cutaneous metastasis was considered unlikely given the localized donor-site distribution, absence of nodular or infiltrative features, and histological absence of malignancy. Nevertheless, the Paracelsus Score, a validated clinical tool for assessing the likelihood of PG (15), yielded 10 points in our patient (threshold ≥10 indicates probable PG), lending additional support to a PG-spectrum diagnosis despite the atypical clinical morphology.

As illustrated in the wound progression (**Figure 1**), the initially unremarkable donor site began to exhibit ICI-associated wound changes. In July 2024, ICI therapy was suspended for 11 weeks, during which the wound showed clinical improvement (**Figure 1E**). Upon reintroduction of the ICI, deterioration set in again despite ongoing topical corticosteroid treatment. After histological diagnosis of a neutrophilic dermatosis, systemic treatment with Dapsone (50 mg twice daily) was initiated after exclusion of contraindications. The patient demonstrated a rapid and marked clinical response already evident at initial follow-up visits, with progressive re-epithelialization and cessation of exudation (**Figures 1I, J**). Gradual tapering of Dapsone is planned under close clinical monitoring.

## 3 Discussion

Neutrophilic dermatoses are a heterogeneous group of inflammatory skin disorders characterized by a sterile accumulation of neutrophils in the skin. PG is among the most recognized entities in this group, typically presenting with rapidly progressing and painful ulcerations (6). Despite its clinical significance, PG remains diagnostically challenging because it lacks specific histopathological features and is frequently mistaken for infected or traumatically complicated wounds (16). The diagnosis is therefore typically made through exclusion of other causes such as infection, malignancy, vasculitis, or drug reactions (10, 11).

Although neutrophilic dermatoses are not uncommon among patients with hematologic malignancies, autoimmune disorders or paraneoplastic phenomena, their emergence as an irAE during ICI therapy appears to be particularly rare (12, 13). Several prior cases offer useful context. One patient with melanoma developed an ICI-induced neutrophilic dermatosis after surgical intervention while being treated with the CTLA-4 inhibitor Ipilimumab. The lesions responded to systemic corticosteroids (17). Another report described a patient with squamous cell carcinoma who developed PG under pembrolizumab, with the lesions responding to systemic corticosteroids (7). Additional reports include cases of PG arising in patients with hepatocellular carcinoma on Atezolizumab, myelodysplastic syndrome under dual ICI therapy and even a gastric cancer patient who developed PG following surgery after completing over five years of well-tolerated Nivolumab therapy. The outcomes in these cases were variable, but in several instances, treatment required escalation beyond corticosteroids to agents such as azathioprine, cyclosporine, and JAK inhibitors (8, 12, 18).

The time to onset in these reported cases has ranged from approximately 12 weeks to over five months after initiation of ICI therapy, with some cases emerging even after cessation of treatment. This variability underscores the unpredictable nature of irAEs and suggests a pattern of lingering immune dysregulation rather than simple direct toxicity. At the mechanistic level, PD-1 blockade may promote neutrophil activation and migration, at least in part through upregulation of CXCL1, CXCL5 and CSF signaling. Notably, neutrophils themselves can express PD-L1, which may play a role in this dynamic (19, 20). Nivolumab in particular has been linked to neutrophil activation in other clinical contexts, lending biological plausibility to this association (9, 21, 22).

The diagnostic classification of our case merits some discussion. Classic PG, EPD, and chronic wound-healing disorder were all considered but none fully accounted for the clinical and histopathological picture on its own. What we observed was a lesion strictly confined to the donor site, with a sterile neutrophilic infiltrate, a pathergy-consistent clinical course, and a clear response to dapsone. Taken together, these features most plausibly support a localized neutrophilic dermatosis on the PG spectrum, arising in the context of PD-1 blockade. We consider this the most accurate diagnostic label available, while acknowledging that boundaries between entities are not always sharp.

In several respects, the clinical course in our patient mirrored prior reports, but also had features we have not seen described elsewhere. The lesion developed specifically at the donor site of a split-thickness skin graft, while the grafted recipient site healed without complication. This discordance raises questions about local tissue susceptibility, pathergy, and microenvironmental differences between the two wound types, though we cannot answer these questions definitely from a single case.

One possible explanation relates to the biology of split-thickness harvesting. Selectively disrupting the epidermis releases damage-associated molecular patterns (DAMPs) and epidermal chemokines, including IL-36, IL-8, and CXCL1, which potently recruit neutrophils (23). Under concurrent PD-1 blockade, this inflammatory cascade may become dysregulated and self-sustaining. By contrast, the recipient site involves primarily deeper dermal tissue and may not trigger the same degree of epidermal DAMP release. The strict confinement of the lesion to the harvest boundaries throughout its entire course is consistent with a pathergy mechanism, the well-described phenomenon whereby cutaneous trauma triggers exaggerated neutrophilic inflammation within a locally sensitized microenvironment. Importantly, the patient consistently reported symptom flares in the days following nivolumab infusions, a pattern that may reflect cyclical immune activation reinforcing this self-sustaining inflammatory loop, though this temporal association does not establish causality.

Site-specific differences in the cutaneous microbiome represent an additional hypothetical contributor to the differential wound response. Substantial variation in microbiome composition across body surface sites is well established (24, 25), and the local microbiome has been shown to modulate immune responses during checkpoint inhibitor therapy (26). However, no microbiome profiling was performed in this case, and this proposed mechanism remains entirely speculative. Systematic studies examining microbiome profiles at graft donor and recipient sites in ICI-treated patients could be informative in the future.

In our case, histopathologic analysis revealed a dense neutrophilic infiltrate in the absence of vasculitis or malignancy, consistent with a neutrophilic dermatosis. Treatment with dapsone, which inhibits recruitment of neutrophils, suppresses the production of superoxide and reactive oxygen species (ROS) as well as elastase release, led to rapid improvement within two weeks (27). This response is consistent with a neutrophil-driven inflammatory process, though it does not independently confirm the underlying etiology.

Importantly, dapsone was the first systemic agent used in this case. Systemic corticosteroids, often first-line for neutrophilic dermatoses like PG, were avoided due to the patient’s medical and therapy history. Prior to initation of dapsone, the patient had completed all 13 cycles of nivolumab and the lesion had demonstrated renewed worsening, with histology confirming a florid ongoing inflammatory process. Sustained re-epithelialization occurred only after dapsone was started, suggesting that discontinuation of ICI alone was insufficient to achieve resolution. Nevertheless, this interpretation rests on a single-case without a rechallenge protocol, and the spontaneous resolution cannot be entirely excluded.

This case illustrates the importance of maintaining high clinical suspicion for rare irAEs like neutrophilic dermatoses in patients undergoing or having recently completed ICI therapy. Chronic, non-healing wounds in this context warrant early biopsy and multidisciplinary evaluation. A neutrophilic dermatosis may present atypically in both morphology and timing, including delayed onset and prolonged course even after therapy cessation (8). Notably, many cases of ICI-associated neutrophilic dermatosis, including our own atypical case, occurred in patients who experienced favorable oncologic outcomes, suggesting a possible link between immune activation and tumor control (7, 18).

### 3.1 Limitations

This case report carries several inherent limitations that are worth stating clearly. As a single case report, it cannot support causal inference and should not be generalized beyond providing hypothesis-generating observations. One significant constraint is the timing of the diagnostic biopsy, which was performed after completion of the one-year immunotherapy course rather than during active nivolumab exposure. Earlier histological sampling would have provided stronger temporal evidence linking PD-1 blockade to the neutrophilic infiltrate. Furthermore, the absence of cytokine and microbiome profiling, and tissue immunophenotyping limits any mechanistic interpretation. Lastly, rechallenge data following the dapsone response are lacking, and long-term follow-up after planned tapering remains to be reported.

### 3.2 Conclusion

This case of an ICI-associated neutrophilic dermatosis reinforces the need for early diagnostic consideration of atypical wounds during or after immunotherapy. It also underscores the relevance of neutrophilic dermatosis as an underrecognized irAE and supports the notion that, even in curative-intent, clinicians must remain vigilant for delayed and atypical adverse events. Timely diagnosis and appropriate immunomodulatory treatment are essential and further studies are needed to elucidate the mechanisms, risk factors, and optimal management of neutrophilic dermatosis in the context of ICI therapy.

## Data Availability

The original contributions presented in the study are included in the article. Further inquiries can be directed to the corresponding author.

## References

[1] Bleiberg BA Gimotty PA Mathew A Selig G Flowers A McClain S . Five-year survival outcomes in patients with melanoma with complete response to immunotherapy who discontinue therapy. J Clin Oncol. (2023) 41:e21503–e. doi: 10.1200/jco.2023.41.16_suppl.e21503 42148471

[2] Robert C . A decade of immune-checkpoint inhibitors in cancer therapy. Nat Commun. (2020) 11:3801. doi: 10.1038/s41467-020-17670-y 32732879 PMC7393098

[3] Keam S Turner N Kugeratski FG Rico R Colunga-Minutti J Poojary R . Toxicity in the era of immune checkpoint inhibitor therapy. Front Immunol. (2024) 15:1447021. doi: 10.3389/fimmu.2024.1447021 39247203 PMC11377343

[4] Brahmer JR Abu-Sbeih H Ascierto PA Brufsky J Cappelli LC Cortazar FB . Society for Immunotherapy of Cancer (SITC) clinical practice guideline on immune checkpoint inhibitor-related adverse events. J Immunother Cancer. (2021) 9. doi: 10.1136/jitc-2021-002435 34172516 PMC8237720

[5] Apalla Z Papageorgiou C Lallas A Delli F Fotiadou C Kemanetzi C . Cutaneous adverse events of immune checkpoint inhibitors: A literature review. Dermatol Pract Concept. (2021) 11:e2021155. doi: 10.5826/dpc.1101a155 33614223 PMC7875661

[6] Marzano AV Borghi A Wallach D Cugno M . A comprehensive review of neutrophilic diseases. Clin Rev Allergy Immunol. (2018) 54:114–30. doi: 10.1007/s12016-017-8621-8 28688013

[7] Tsibris H Lian C Ho A . Pembrolizumab-associated pyoderma gangrenosum in a patient with metastatic squamous cell carcinoma. Dermatol Online J. (2021) 27. doi: 10.5070/d3274053158 33999581

[8] Sato M Kawai K Yamada A . Postoperative pyoderma gangrenosum in a patient undergoing long-term nivolumab therapy. J Cutaneous Immunol Allergy. (2024) 7:2024. doi: 10.3389/jcia.2024.13751

[9] Ravi V Maloney NJ Worswick S . Neutrophilic dermatoses as adverse effects of checkpoint inhibitors: A review. Dermatol Ther. (2019) 32:e13074. doi: 10.1111/dth.13074 31444856

[10] Dissemond J Marzano AV Hampton PJ Ortega-Loayza AG . Pyoderma gangrenosum: Treatment options. Drugs. (2023) 83:1255–67. doi: 10.1007/s40265-023-01931-3 37610614 PMC10511384

[11] George C Deroide F Rustin M . Pyoderma gangrenosum - a guide to diagnosis and management Clin Med (Lond). Clin Med (Lond). (2019) 19:224–8. doi: 10.7861/clinmedicine.19-3-224 PMC6542232 31092515

[12] Welborn ME Kubicki SL Patel AB . Pyoderma gangrenosum following initiation of immune checkpoint inhibitor therapy. J Immunotherapy Precis Oncol. (2020) 1:82–4. doi: 10.4103/jipo.jipo_11_18 42303403

[13] Kridin K Ankary-Khaner M Kridin M Cohen AD Badarny S . Hematological Malignancy-associated pyoderma gangrenosum: Evaluating the magnitude of the association. Front Med. (2024) 11:2024. doi: 10.3389/fmed.2024.1425454 39118665 PMC11306151

[14] Patton D Lynch PJ Fung MA Fazel N . Chronic atrophic erosive dermatosis of the scalp and extremities: A recharacterization of erosive pustular dermatosis. J Am Acad Dermatol. (2007) 57:421–7. doi: 10.1016/j.jaad.2007.04.026 17532096

[15] Jockenhöfer F Wollina U Salva KA Benson S Dissemond J . The PARACELSUS score: A novel diagnostic tool for pyoderma gangrenosum. Br J Dermatol. (2019) 180:615–20. doi: 10.1111/bjd.16401 29388188

[16] Owen BS Pacult MA Lee BS . Pyoderma gangrenosum masquerading as wound infection in the early postoperative period after lumbar spine deformity correction surgery. Cureus. (2022) 14:e25545. doi: 10.7759/cureus.25545 35800799 PMC9246455

[17] Rudolph BM Staib F Von Stebut E Hainz M Grabbe S Loquai C . Neutrophilic disease of the skin and intestines after ipilimumab treatment for Malignant melanoma - simultaneous occurrence of pyoderma gangrenosum and colitis. Eur J Dermatol. (2014) 24:268–9. doi: 10.1093/milmed/103.5.359 24721740

[18] Kim HS Kwon JE Park YJ . Atezolizumab plus bevacizumab-induced recalcitrant pyoderma gangrenosum treated with baricitinib: A case report. Acta Derm Venereol. (2023) 103:adv9646. doi: 10.2340/actadv.v103.9646 37526292 PMC10413870

[19] Cristinziano L Modestino L Capone M Madonna G Mallardo D Giannarelli D . PD-L1(+) neutrophils as novel biomarkers for stage IV melanoma patients treated with nivolumab. Front Immunol. (2022) 13:962669. doi: 10.3389/fimmu.2022.962669 36016960 PMC9398490

[20] Roudko V Del Valle DM Radkevich E Kelly G Hui X Patel M . Immunological biomarkers of response and resistance to treatment with cabozantinib and nivolumab in recurrent endometrial cancer. J Immunother Cancer. (2025) 13. doi: 10.1136/jitc-2024-010541 40010771 PMC11865721

[21] Rovedatti L Lenti MV Vanoli A Feltri M De Grazia F Di Sabatino A . Nivolumab-associated active neutrophilic gastritis. J Clin Pathol. (2020) 73:605–6. doi: 10.1136/jclinpath-2020-206540 32161070

[22] Lamberg O Pandher K Matthews NH . Nivolumab-induced hidradenitis suppurativa: A case report. Dermatol Online J. (2024) 30. doi: 10.5070/d330464106 39644467

[23] Piipponen M Li D Landén NX . The immune functions of keratinocytes in skin wound healing. Int J Mol Sci. (2020) 21. doi: 10.3390/ijms21228790 33233704 PMC7699912

[24] Grice EA Kong HH Conlan S Deming CB Davis J Young AC . Topographical and temporal diversity of the human skin microbiome. Science. (2009) 324:1190–2. doi: 10.1126/science.1171700 19478181 PMC2805064

[25] Grice EA Segre JA . The skin microbiome. Nat Rev Microbiol. (2011) 9:244–53. doi: 10.1038/nrmicro2537 21407241 PMC3535073

[26] Gopalakrishnan V Spencer CN Nezi L Reuben A Andrews MC Karpinets TV . Gut microbiome modulates response to anti-PD-1 immunotherapy in melanoma patients. Science. (2018) 359:97–103. doi: 10.1126/science.aan4236 29097493 PMC5827966

[27] Rakočević S Mališ V Kozić L Dubovina A Drakul M Bokonjić D . Dapsone alters phenotypical and functional properties of human neutrophils *in vitro*. Molecules. (2025) 30:113. doi: 10.3390/molecules30010113 PMC11722540 39795170

